# Platelet Dysfunction in Blood Donors Detected by Platelet Function Analyzer PFA-100™

**DOI:** 10.7759/cureus.25497

**Published:** 2022-05-30

**Authors:** Malika Belkacemi, Abdellah Berber

**Affiliations:** 1 Hemobiology and Blood Transfusion, “Hassani Abdelkader” University Hospital, Sidi Bel Abbès, DZA; 2 Faculty of Medecine, University Djillali Liabes, Sidi Bel Abbès, DZA; 3 Central Laboratory, “Hassani Abdelkader” University Hospital, Sidi Bel Abbès, DZA

**Keywords:** von willebrand factor, blood group o, aspirin-like defect, platelet function test, blood donors

## Abstract

Background

Platelet transfusions may be indicated to prevent and treat bleeding in patients with quantitative or qualitative platelet defects. Millions of platelet components are transfused worldwide. It is well known that platelet dysfunction predicts blood loss after surgery. Hence, the quality of the platelet donation and the resulting platelet concentrate are critical for the transfusion. The aim of this study is to assess platelet function in well-qualified blood donors.

Methodology

Blood samples from 275 blood donors were collected in 0.129 M (3.8%) sodium citrate tubes prior to routine blood donation. Platelet function was assessed by measuring the closure time (CT) on the platelet function analyzer (PFA-100™; Siemens Health Diagnostics, Marburg, Germany) using collagen/epinephrine (CEPI) and collagen/ADP (CDP) cartridges.

Results

Using the PFA-100™, 20.4% of donors had an abnormal platelet function test, of whom 9.4% had prolonged CT with two cartridges, 7% had only prolonged CEPI CTs consistent with aspirin-like defect, and 4% had prolonged CADP CTs only. We found no closure (>300 seconds) in 6.54% of donors, including 1.45% with the CEPI cartridge, 2.9% with the CADP cartridge, and 2.18% with CEPI and CADP cartridges. Level of von Willebrand factor ristocetin cofactor (vWF: RCo) activity was 112% (56-168%). Of the factors examined (age, sex, cigarette smoking, blood donation type, ABO, and Rhesus blood group), only blood group O was significantly linked with impaired platelet function test in qualified blood donors (p = 0.023; odds ratio = 1.981; 95% confidence interval (1.091-3.595)).

Conclusions

Some qualified blood donors present abnormal platelet function results. More research is required to provide greater insight into the impact of platelet dysfunction in blood donors on the clinical efficacy of their platelet components. This study has confirmed that the influence of ABO blood group on the CT PFA-100™is not wholly dependent on vWF.

## Introduction

Platelets are small discoid enucleated circulating blood cells. When they recognize damaged blood vessel walls, they bind together to stop and avoid bleeding. Platelet transfusions may be indicated to prevent and treat bleeding in patients with quantitative or qualitative platelet defects. Millions of platelet components are transfused worldwide. More than 50% of these components are transfused to patients undergoing chemotherapy or radiotherapy, where they have become an integral part of their therapy [1]. It is well known that platelet dysfunction predicts blood loss after surgery. Therefore, the platelet quality of individual donations is critical for transfusion. In the current Algerian practice, platelet donors do not undergo platelet function testing. Normal platelet function is defined as an absence of a history of bleeding through a routine questionnaire and physical examination. However, this procedure may fail to detect acquired platelet defects in donors. Although the platelet storage lesion has been well characterized, the influence of donors on platelet function has rarely been studied. The lack of research in this area reflects that no simple, reliable, and quick assessment was available for testing platelet function in the past.

Recently, there has been significant progress in assessing platelet function testing, leading to simpler tests that can be used outside of specialized hemostasis laboratories. One of these tests is a platelet function analyzer (PFA) test which simulates a high shear-dependent platelet function. Citrated whole blood passes at high shear stress through disposable cartridges containing an orifice in a membrane covered with collagen and epinephrine (CEPI) or collagen and ADP (CADP). Under high shear stress, these agonists induce a plug formation in and around the orifice. The time it takes for the orifice to close and stop blood flow is referred to as the closure time (CT).

The test is easy to perform, fast (maximum CT is 300 seconds), and can test small volumes of citrated blood (800 µL/cartridge) within four hours of collection. Yet, there are several variables that may affect PFA test results. CT depends on citrate concentration, blood collection time, plasma von Willebrand factor (vWF) level, ABO blood group, some drugs, and certain foods [2]. In addition, a low platelet count or low hematocrit can prolong CT. The PFA device is now available in clinical laboratories. This study aimed to assess platelet function in well-qualified blood donors.

## Materials and methods

Selection of donors and study design

We performed this cross-sectional study at the blood transfusion department of Sidi Bel Abbès University Hospital. We conducted this study for 11 months from December 2018 to October 2019. Donors who reported for blood donation were invited to participate, and all gave consent. We selected subjects based on the inclusion and exclusion criteria as recommended by national guidelines for the selection of blood donors. After a physical examination and routine questionnaire, we included subjects who denied regular or recent aspirin intake or another cyclooxygenase inhibitor. We also recorded the ABO and Rhesus blood groups of each donor. Exclusion criteria were platelet count below 100 G/L or hematocrit <0.30 and vWF level <50%. The study was approved by the Ethics Committee of Sidi Bel Abbès University Hospital (CE/CHU/15/2017). A total of 283 donors participated in the study. We excluded eight donors because of the intake of acetylsalicylic acid (ASA) or non-steroidal anti-inflammatory drugs (NSAIDs).

Donor sampling and processing

Prior to blood donation, blood samples were taken from the donor using a blood donation vacuum. Blood was drawn into ethylenediaminetetraacetic acid (EDTA) tubes for the determination of complete blood counts (CBCs) and two 0.129 M (3,8%) citrate tubes to measure CTs and vWF levels. The samples for PFA testing were stored at room temperature before testing. The PFA test was performed less than four hours after venipuncture, as recommended by the manufacturer. Platelet-poor plasma (PPP) was obtained by centrifugation at 3500× g for 10 minutes. Aliquots of 0.5 mL of PPP were snap-frozen and stored at -80°C. When required for further analysis, vWF samples were thawed at 37°C for 10 minutes. Hemolyzed or clotted samples were not used.

Assays

Complete Blood Count (CBC)

The CBC was performed using the Sysmex XT-2000i Automated Hematology Analyzer (Sysmex Corporation, Kobe, Japan).

*PFA-100™*​* Test*

PFA CEPI CT and CADP CT were measured on a PFA-100™ instrument from Siemens Health Diagnostics (Marburg, Germany) using a single lot for each test cartridge.

Von Willebrand Factor Ristocetin Cofactor Activity (VWF:RCo)

VWF:RCo was determined using a manual visual agglutination assay with BC von Willebrand Reagent (Siemens, Marburg, Germany).

Definition of the cases

According to the local reference intervals of CT PFA-100™, platelet dysfunction was defined by the CEPI CT of >216 seconds or the CADP CT of >148 seconds [3]. The maximum value for the CT is 300 seconds, as provided by the manufacturer. Values greater than 300 seconds are reported as non-closure, and in this case, a value of 301 seconds was arbitrarily assigned for further conservative statistical comparisons. The aspirin-like defect was characterized by an abnormal CEPI CT and normal CADP CT.

Data analysis

The Kolmogorov-Smirnov test of normality was used to analyze the values. Continuous variables are expressed as the mean and standard deviation (SD), or median and range in the case of non-normally distributed data. The t-test or the Mann-Whitney U-test was used to compare groups. Moreover, qualitative variables were reported as numbers or percentages with 95% confidence intervals (CIs). Either the chi-square test or the Fisher test was used to check frequency difference. We performed statistical analysis using the Statistical Package for Social Sciences (SPSS) version 25 (IBM Corp., Armonk, NY, USA) and MedCalc Statistical Software version 15 (MedCalc Software, Ostend, Belgium). For all statistical tests, the level of signiﬁcance was set at a p-value of 0.05.

## Results

A total of 275 qualified blood donors were recruited. The male/female sex ratio was 3.4. Most donors (79.3%) donated whole blood, while 20.7% donated apheresis platelets. The distribution of participants by ABO Rh blood group was as follows: 45.5% were O, 30.2% were A, 19.3% were B, 5.1% were AB, and 88% were Rh-positive. Because we know the O, A, B, and AB phenotypes in the Algerian population are 44.04%, 39.28%, 12.84%, and 3.84%, respectively, there is no difference in the blood groups [4]. Baseline data for all blood donors, male and female, are set out in Table 1. There is no age difference between women and men. Moreover, there was no significant difference in the proportion of blood group O and the vWF level between the genders. However, there appears to be a substantial difference between the groups in terms of blood type donation. As expected, the proportion of women smokers was significantly lower than that of men smokers.

**Table 1:** Baseline data of 275 qualified blood donors. Chi-square test. *Student’s test; ^†^Mann-Whitney U test. Data are expressed as median value (minimum-maximum) and arithmetic mean ± SD or number. NS: non-significant difference; SD: standard deviation; WBC: white blood cell; RBC: red blood cell; Hct: hematocrit; Hb: hemoglobin; RH: Rhesus; MPV: mean platelet volume; vWF:RCo: von Willebrand:Ristocetin cofactor activity

	All	Male	Female	P-value
Number of subjects	275	224	51	
Age (years)	32 (19–60)	33 (19–60)	26 (19–55)	NS
Whole blood donor/Platelet pheresis	218/57	172/52	46/5	0.03
Smokers/Non-smokers	70/205	69/155	1/50	<0.0001
Blood group O/Non-O blood group	125/150	103/121	22/28	NS
Rh-positive/Rh-negative	242/33	196/28	46/5	NS
WBC (G/L)*	6.81 (1.71)	6.86 (1.73)	6.57 (1.62)	NS
RBC (T/L)*	4.87 (0.48)	4.99 (0.41)	436 (0.42)	<0.0001
Hb (g/dL)^†^	14.8 (11–17.5)	15 (12.3–17.5)	12.7 (11.0–15.5)	<0.0001
Hct (%)^†^	437 (31.8–51.1)	44.2 (31.8–51.1)	38.1 (31.9–46.2)	<0.0001
Platelet count (G/L)	211 (140–453)	208 (140–453)	218 (142–365)	NS
MPV fL*	10.5 (0.97)	10.5 (0.98)	10.7 (1.04)	NS
vWF:RCo^† ^(%)	112 (56–168)	112 (56–168)	112 (56–168)	NS

**Figure 1:**
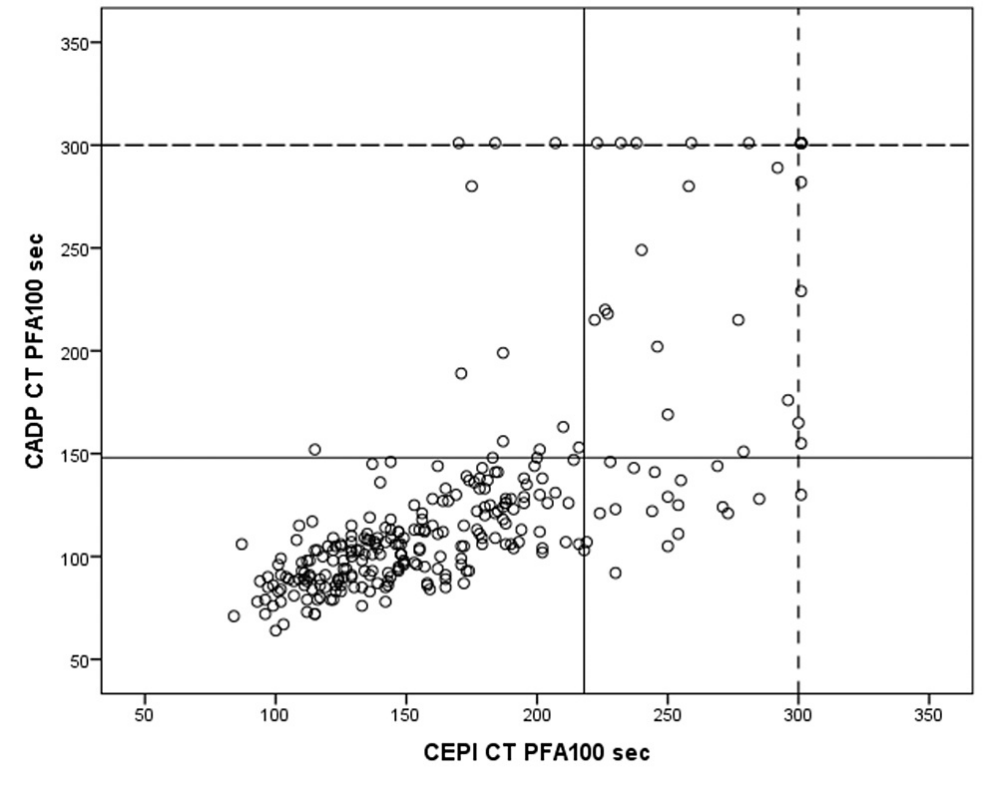
Distribution of closure time PFA-100TM among blood donors. The solid line indicates the upper limit of closure time. The dotted line indicates a closure time of >300 seconds.

Overall, 20.4% of donors (56 of 275) had abnormal platelet function tests, of which 9.4% had prolonged CT with two cartridges, about 7% had only long CEPI CTs, which showed an aspirin-like profile, and 4% had only long CADP CTs. We reported no closure (>300 seconds) in 6.5% of qualified donors, including 1.4% with CEPI, 2.9% with CADP, and 2.2% with both cartridges (CEPI and CADP). These results are shown in Figure 1.

Table 2 compares the results obtained from the current study and other reports. As can be seen from the table, there was no observed difference in the percentage of platelet dysfunction.

**Table 2:** Rate of platelet dysfunction in blood donors from three reports. CI: confidence interval; NS: non-significant difference; CT: closure time; CEPI: collagen and epinephrine

	Current study	Jilma-Stohlawetz et al. 2001 [5]	Harrison et al. 2004 [6]	Paglieroni et al. 2004 [7]
Country	Algeria	Australia	United Kingdom	United States
Number of participants	275	206	100	24
Donation type	Whole blood and plateletpheresis	Plateletpheresis	Plateletpheresis	Plateletpheresis
Method	PFA100™	PFA100™	PFA100™	PFA100™
Rate of platelet dysfunction (%) (95% CI)	20.4 (17.6–23.2)	20 (14.5–25.5)	24 (15.6–32.4)	46 (26–66)
P-value		NS	NS	NS
An aspirin-like defect (%) (95% CI)	7 (1.6–12.4)	13.6 (8.9–18.3)	16 (8.8–23.2)	38 (19–57)
P-value		NS	NS	NS
CT CEPI only >300 seconds (%) (95% CI)	1.4 (0.9–1.9)	11 (9.2–12.8)	4 (3.5–5.5)	17 (11–18)
P-value		NS	NS	NS

Of the factors examined, we found that blood group O was linked with impaired platelet function in qualified blood donors. These ﬁndings are presented in Table 3.

**Table 3:** Variables associated with impaired platelet function in blood donors. OR: odds ratio; CI: confidence interval; NS: non-significant difference

Variable	OR	95% CI	P-value
Age (<41 years)	1.285	0.665–2.481	NS
Sex (F/M)	1.259	0.609–2.602	NS
Blood donation type	1.364	0;684–2.722	NS
Cigarette smoking	1.376	0.740–2.557	NS
Blood group O	1.981	1.091–3.595	0.023
RH blood group	1.293	0.594–3.049	NS

## Discussion

This study was conducted to assess the function of platelets obtained from blood donors. We noted abnormal platelet function tests in up to 20% of qualified blood donors. This observation pattern has been shown in previous studies [5-7]. This finding confirmed that impaired platelet function test using PFA100™ is common in blood donors. Thus, in the surgical setting, these defects may lead to bleeding and can explain the transfusion inefficiency of some PCs.

There was no notable sex and age difference in the platelet dysfunction rate. Our study findings confirm previous observations that CT is not influenced by sex and age [8-10].

The results showed that platelet function was not influenced by blood donation type. Moreover, it appears that the apheresis procedure is stressful for some donors. It is well known that anxiety may disrupt platelet function. Yet, it has been demonstrated that the quality of apheresis platelets is not affected by the anxiety levels of blood donors [11]. Thus, these findings further support the results of the present study.

Our findings revealed that blood group O was significantly associated with platelet dysfunction. This relationship was further supported in a recent study, which showed that blood group O is a risk factor for increased bleeding, independent of vWF and FVIII levels [12]. However, the exact underlying mechanism is not clear. Our results confirmed a recent publication that the effect of the ABO group on PFA-100™ CT is not wholly dependent on vWF [13].

In this study, we observed that the incidence of prolonged CT in smokers was comparable to that in non-smokers. Our study findings support that smoking does not significantly change the quality of platelets [14]. Thus, the current practice of not excluding smokers from platelet donation can be continued.

Around 7% of blood donors showed an aspirin-like defect. These findings are similar to those reported previously [5-7]. We know that platelet donors are deferred if they have taken aspirin, as required by transfusion guidelines. Other blood components can be drawn from a donor who has taken aspirin. It has been shown that 7% of donors had consumed aspirin [15]. This finding stems from the fact that aspirin is a widely used over-the-counter drug and many donors may not think of it as a drug. Moreover, some donors may not remember taking aspirin. We know that aspirin irreversibly inhibits platelet cyclooxygenase (COX). Hence, the effect of aspirin on platelets can persist throughout the lifespan. This effect leads to blocking the synthesis of thromboxane A2 (TXA2) in platelets.

It should be noted that TXA2 is a key platelet feedback agonist. However, it has been shown that a large number of donors with severe prolongation of CT CEPI had levels of TXB2 suggesting no use of aspirin [5]. Moreover, the most prolonged CT CEPI is transient. Thus, aspirin is not the only cause for the prolongation of CEPI CT. We also found a non-negligible proportion (9.4%) of donors with platelet defects due to prolonged CTs on both cartridges and a minority of donors (4%) with only prolonged CT CADP. Other donor studies also reported similar results. As assessed by in vitro studies, platelet function is affected by various factors. According to a literature search, it is clear that factors related to blood donors are mainly dietary factors and some drugs [16]. The effect of diet and drugs on platelets, particularly platelet donors, will be discussed next.

Other than aspirin, NSAIDs cause impaired platelet function. In contrast to aspirin, these agents reversibly inactivate COX. Their effect on platelets is thought to last up to a week. It has been shown that the effect of ibuprofen, a common NSAID, on platelet function, as measured by the PFA-100™,returns to normal within 24 hours of ceasing the drug [17]. NSAIDs, including aspirin, are commonly used and not thought of as a drug by some donors. Thus, such drug intake may not be declared prior to donation.

In addition to NSAIDs, including aspirin, many drugs have been shown to affect platelet function in vitro. Yet, the clinical relevance of this inhibition remains unclear. These drugs include antibiotics (very high doses of penicillin), cardiovascular drugs, thrombolytic drugs, and chemotherapy drugs. In the case of blood donation, donors must be healthy. Hence, people taking these medications are excluded from blood donation.

Based on ex vivo or in vitro studies, many components of commonly used foods have been proposed to alter platelets. However, the clinical consequence of diet-induced platelet defects is not clear. Further, the impact of these defects on the efficacy of transfusion of platelets is unknown. The duration of the effect on platelets is also unclear for many of these components. However, some components appear to temporarily alter platelets. Blood donors are advised to eat prior to donation, and although the effects of these components disappear within hours, some donors can donate ex vivo defective platelets. As noted in this study, two of twenty-four whole blood donors had CEPI CT of ≥300 seconds, which they attributed to the consumption of chocolate six hours prior to donation [7].

Inhibition of the TXA2 pathway appears to be a common target for many food components. It has been reported that the compound ginger inhibits platelet aggregation in vitro to decrease the thromboxane B2 level [18]. A diet rich in fish or marine oil can slightly prolong bleeding time. Marine fats have been shown to reduce platelet function by competing with arachidonic acid (AA) for COX [19]. Onion has been shown to have antiaggregatory effects by altering AA metabolism in platelets [20]. It has been shown that consuming flavonoid-rich foods such as green tea and grape juice or red wine and cocoa may inhibit platelet aggregation [21]. Numerous mechanisms for this inhibition have been proposed. There is a blocking of the thromboxane receptor by some flavonoids and interference with the TXA2 production pathway by inhibiting both AA liberation and TXA2 synthesis. Components involved in these mechanisms can produce an aspirin-like defect when stimulated with AA. Moreover, similar aspirin patterns have been noted on the PFA-100™ test after consumption of cocoa and chocolate [22,23]. It is now well established that prolongation of CEPI CT in the PFA-100™ test predicts bleeding during surgery [24]. Yet, it is unknown whether such diets are associated with an increased risk of clinically important bleeding.

Other mechanisms of inhibition of platelet function by some diet and dietary elements have been identified. Garlic mainly inhibits platelet function via the inhibition of COX-1, similar to aspirin, and the direct interaction with fibrinogen receptors [25]. Soya isoflavonoids showed marked platelet activation suppression through the impairment of major platelet signaling patterns [26]. Consequently, eating these food components may account for the CT PFA-100™prolongation on both cartridges. Furthermore, CADP CT has been shown to be prolonged for long hours after dark chocolate consumption [27]. Cocoa flavanols have been shown to exert antioxidant properties by directly increasing nitric oxide (NO) levels [28]. It is clear from previous research that NO-dependent platelet activation is more strongly targeted by ADP than epinephrine in healthy subjects [29,30]. The mechanism behind this may explain why some studies failed to observe the prolongation of CEPI CT on PFA-100™after consuming dark chocolate.

Donor-related platelet defects may undermine the effectiveness of platelet therapy. Although it appears that these factors might affect the quality of PCs, there is still significant work that needs to be done to understand their impact on the efficacy of platelet transfusion.

There were some strengths and limitations of this research. The sample size is a key strength of the present study. Our sample size was much larger than previous similar studies. To our knowledge, this study is the first to access platelet function in Algerian blood donors. This study has all of the limitations linked with a retrospective study. The main limitation comes from the non-randomized selection of the sample. Despite this limitation, the findings of this research are important because they have highlighted several questions that need further investigation.

## Conclusions

This study set out to assess platelet function in Algerian blood donors, as studied using PFA-100™. The most obvious finding of this survey is that a number of donors present with prolonged CT. Yet, more research is needed to obtain greater insight into the impact of platelet dysfunction in blood donors on the clinical efficacy of their platelet components. Moreover, this study has confirmed the finding of recent research, which found that the influence of the ABO blood group on the CT PFA-100™ is not entirely dependent on vWF. This study also points to the need for more work to be done to elucidate the exact mechanism of the association of these factors. The overall findings highlight a fruitful area for further investigation.
